# Cardiac adaptations to patent ductus arteriosus ligation in preterm infants: a speckle-tracking study

**DOI:** 10.1038/s41390-025-04182-y

**Published:** 2025-07-19

**Authors:** Katsuaki Toyoshima, Hirosato Aoki, Takahiro Noguchi, Naka Saito, Tatsuto Shimizu, Gakuto Ujiie, Takahiro Kemmotsu, Yusuke Morita, Tomoyuki Shimokaze, Tomoko Saito, Jun Shibasaki, Motoyoshi Kawataki, Yurika Furukawa, Toshihide Asou, Tsuyoshi Tachibana, Satoshi Masutani

**Affiliations:** 1 Department of Neonatology, Kanagawa Children’s Medical Center, Yokohama, Japan; 2 Department of Clinical Laboratory, Kanagawa Children’s Medical Center, Yokohama, Japan; 3 Department of Cardiovascular Surgery, Kanagawa Children’s Medical Center, Yokohama, Japan; 4 Department of Pediatrics, Saitama Medical Center, Saitama Medical University, Kawagoe, Japan

## Abstract

**Background:**

Understanding the complex cardiac adaptations following patent ductus arteriosus (PDA) ligation in preterm infants is essential for optimizing postoperative care. This study tested the hypothesis that left ventricular (LV), left atrial (LA) and right ventricular (RV) longitudinal strain are acutely impaired and then recover after PDA surgery in preterm infants.

**Methods:**

Thirty-two preterm infants who underwent PDA ligation (median gestational age: 25 weeks) were evaluated using speckle-tracking echocardiography to quantify LV, RV, and LA longitudinal strain before and at 4–8 and 24–48 h post-surgery. These data were compared with 36 preterm infants without PDA (non-PDA).

**Results:**

LV global longitudinal strain (LVGLS) was higher and RV free wall longitudinal strain (RVFWSL) was lower in the PDA group than in the non-PDA group preoperatively (both p < 0.01). In the non-PDA group, LVGLS and RVFWSL were −16% and −28%, respectively. In the PDA group, LVGLS at the three time points was −20%, −13%, and −15%, and RVFWSL was −21%, −21%, and −25%, respectively (all p < 0.05). LA reservoir strain (LASr) initially decreased and then increased.

**Conclusions:**

After PDA ligation, LVGLS and LASr transiently decrease then increase within 24–48 h, while RVFWSL normalizes without a decrease.

**Impact:**

- Preoperatively, preterm infants with PDA had higher left ventricular global longitudinal strain (LVGLS), and lower right ventricular free wall strain (RVFWSL) than those without PDA.
- After PDA ligation, LVGLS and left atrial reservoir strain (LASr) initially decreased and then recovered within 24–48 h, while RVFWSL rapidly improved.
- Our findings indicate dynamic and different post-surgical cardiac adaptation in the right ventricle and left ventricle after PDA ligation.

## Introduction

Patent ductus arteriosus (PDA) is one of the major complications of preterm infants^1^. With a decrease in pulmonary resistance after birth, PDA may cause a larger left-to-right shunt throughout the cardiac cycle, causing high pulmonary flow and ischemia of systemic organs. Therefore, instability induced by PDA is associated with intraventricular hemorrhage or necrotizing enterocolitis. Moreover, regardless of whether there is surgical or percutaneous closure, closure of PDA causes an abrupt change in left ventricular (LV) loading conditions. In this situation, preterm infants are at a high risk of hemodynamic compromise, leading to hypotension, reduced cardiac output, and compromised oxygenation and ventilation^2–4^ after the closure of PDA^5^. Approximately 10% to 45% of preterm infants undergoing surgical PDA ligation encounter post-ligation hemodynamic instability, which may affect their long-term outcomes^6^.

However, how hemodynamic instability occurs following the surgical closure of PDA remains unclear. Several studies that used conventional echocardiography reported the changes in LV function associated with an increase in LV afterload induced by PDA closure^3,4,7–11^. Such instability in the LV after PDA closure and preoperative excessive preload in the LV and left atrium (LA) suggest that PDA is a disease of the left heart system. Our recent study using three-dimensional (3D) echocardiography showed that the right ventricular (RV) volume was significantly larger and the RV ejection fraction (EF) was significantly smaller in preterm infants before PDA surgery^12–14^. These findings suggest that the function of not only the LV and LA, but also the RV, is crucial in preterm infants with PDA.

Assessment of LV, LA, and RV function using speckle-tracking echocardiography has recently become a standard clinical tool for the evaluation of cardiac function in adults. Longitudinal strain analysis using speckle-tracking echocardiography equipped with artificial intelligence provides a simple, reproducible, and promising echocardiographic evaluation for the early detection of cardiac dysfunction and heart failure. LV global longitudinal strain (LVGLS) measured by speckle-tracking echocardiography is increasingly being recognized as a more effective technique than the conventional LV ejection fraction (LVEF) in detecting subtle changes in ventricular function and in predicting outcomes^15,16^. Longitudinal strain of the LA and RV measurements have also been standardized and are being used to assess cardiac function^17–21^. The longitudinal strain assessment of LV, LA, and RV using speckle-tracking echocardiography has also been sporadically reported in preterm infants^22–30^. However, in preterm infants with PDA, previous studies using speckle-tracking echocardiography have assessed the LV^27,28^ and LA^23,25^, but not the RV. Our previous study briefly described the values of GLS and global circumferential strain measured by 3D echocardiography for the LV and LA as a ref. ^14^. Our institution initiated the use of AutoStrain (AutoStrain LV/LA/RV; TomTec Imaging Systems, Unterschleissheim, Germany), which is a machine learning-based speckle-tracking software, during the middle of the observation period of our previous study^14^, using 3D echocardiography.

Although we measured LVGLS using 3D echocardiography in our previous study, its lower frame rate and different computational principles compared with 2D speckle-tracking echocardiography make direct comparisons challenging. The use of AutoStrain may provide further insight in myocardial longitudinal strain and essential reference values for LV, RV or LA longitudinal strain variables in preterm infants. This software may allow standardized and reproducible assessment of myocardial strain in preterm infants, and may help identify functional differences between those with severe PDA requiring surgical ligation and those without PDA.

This study aimed to test the hypothesis that not only LVGLS, but also LA longitudinal strain (reservoir, conduit, and contraction) and RV free wall longitudinal strain (RVFWSL), may be acutely impaired and then recover after PDA surgery in preterm infants as shown by speckle-tracking echocardiography using artificial intelligence.

## Methods

### Study design and population

This retrospective study, which was conducted at a single center, enrolled preterm infants with a gestational age ranging from 23 to 33 weeks who underwent PDA surgery and echocardiographic evaluation including AutoStrain between November 2019 and December 2022. We excluded preterm infants with (1) cardiac anomalies other than a patent foramen ovale and a persistent left superior vena cava, (2) chromosomal abnormalities, and (3) apparent clinical syndrome or multiple abnormalities. These patients who underwent PDA surgery were also included in our previous study^14^.

Referrals for PDA ligation were triaged on the basis of clinical and echocardiographic findings. The indications for surgical PDA closure were as follows: (1) difficulty in weaning from mechanical ventilation; (2) worsening congestive heart failure despite medical management or contraindication to cyclooxygenase inhibitors; (3) a transductal diameter > 1.5 mm, predominantly left-to-right flow; (4) LA enlargement as indicated by an LA/aortic (LA/Ao) diameter ratio > 1.3 or LA volume index > 1.0 ml/kg; and (5) left pulmonary artery end-diastolic velocity (LPAedv) > 15 cm/s on echocardiography. Speckle-tracking echocardiography has been part of our standard protocol since November 2019. The indication for PDA surgery was determined by neonatologists who were blinded to the speckle-tracking echocardiographic data. LV, LA, and RV function in patients who underwent PDA ligation (PDA group) were evaluated by transthoracic echocardiography within 12 h before PDA ligation, within 4–8 h after PDA ligation, and between 24 and 48 h postoperatively.

Preoperative LVGLS, RVFWSL, RV four-chamber longitudinal strain including the ventricular septum (RV4cSL) and phase-specific LA longitudinal strain (LA contraction [LASct], conduit [LAScd], and reservoir [LASr]) in preterm infants in the PDA group were compared with those in preterm infants without PDA (non-PDA group). The inclusion criteria of the non-PDA group were as follows: (1) 14-day-old neonates with a gestational age between 23 and 28 weeks and those with a gestational age between 29 and 31 weeks who required mechanical ventilation who were admitted to our neonatal intensive care unit between October 2020 and December 2022; (2) preterm infants who did not have PDA ligation; (3) PDA closure before day 14; and (4) preterm infants who did not have any congenital heart diseases or pulmonary hypertension as indicated by a non-circular LV shape at the peak of systole and/or tricuspid regurgitation pressure gradient > 32 mmHg^31^. An echocardiography dataset including speckle-tracking echocardiography was acquired in all preterm infants with these criteria as part of the institutional protocol.

This study was conducted in accordance with the principles contained in the Declaration of Helsinki and was approved by the institutional review board of Kanagawa Children’s Medical Center (No. 1806-07).

### Clinical characteristics

Data of the gestational age in weeks, birth weight, sex, Apgar scores, age (days) at PDA surgery, corrected gestational age in weeks, and body weight at the surgery day were collected from medical records. In the PDA group, details of the treatment received, survival or death at discharge, and additional characteristics were obtained.

All data, comprising respiratory characteristics, baseline hemodynamics, and echocardiograms, were collected at the three specified time points. N-terminal pro-brain natriuretic peptide concentrations were quantified at the pre-ligation stage and at 24–48 h following surgical intervention as part of our standard protocol.

The collected clinical data included heart rate, oxygen saturation, the fraction of inspired oxygen, mean airway pressure, and blood pressure. Blood pressure was measured immediately before an echocardiogram. Postoperative blood pressure at all time points in the PDA group was measured using an arterial line. Blood pressure was measured using the oscillometer technique in all preterm infants in the non-PDA group and at certain preoperative points in those in the PDA group who did not have an arterial line.

When the echocardiogram was performed, the respiratory severity score was calculated as the mean airway pressure (mmHg) × the fraction of inspired oxygen^32^. The vasoactive–inotropic score at the initial echocardiographic examination was calculated as the dopamine dose (μg/kg/min) + the dobutamine dose (μg/kg/min) + 100 × the epinephrine dose (μg/kg/min) + 100 × the norepinephrine dose (μg/kg/min) + 10,000 × the vasopressin dose (U/kg/min) + 10 × the olprinone dose (μg/kg/min)^33^.

### Speckle-tracking echocardiography

**Fig. 1:**
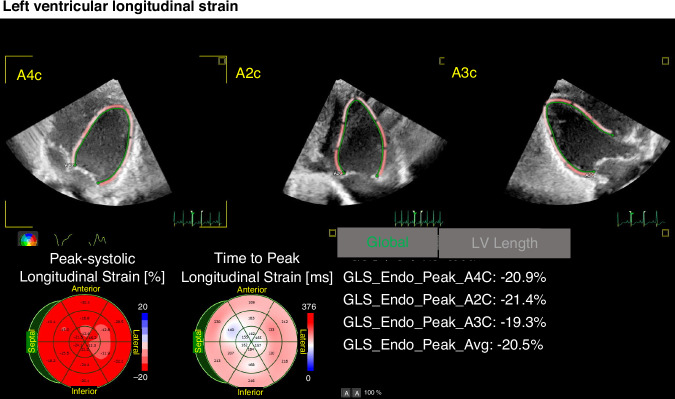
Analysis of LV longitudinal strain by speckle-tracking echocardiography. *A4C* apical four-chamber view, *A2C* apical two-chamber view, *A3C* apical three-chamber view*, GLS* global longitudinal strain*, LV* left ventricular, *Endo* endocardial*, Avg* average of A4, A2C, andA3C.

An experienced echocardiographer (K. Toyoshima) performed the echocardiography with an ultrasound device (EPIC 7 G or EPIQ CVx with an S9-2 probe; Philips Healthcare, Andover, MA). As part of the protocol, speckle-tracking echocardiography was used to assess LVGLS, RVFWSL, RV4cSL, LASct, LAScd, and LASr. Speckle-tracking analysis was performed by the same observer (K. Toyoshima) using AutoStrain LV/LA/RV. Two-dimensional images were acquired from apical four-chamber (A4C), apical two-chamber (A2C), and apical three-chamber (A3C) views for LV longitudinal distortion analysis. Frame rates of 80–100 frames/s were used for storage and analysis. AutoStrain is an automated strain measurement application equipped with artificial intelligence integrated into the Philips ultrasound system. This software automatically detects and labels selected apical views and applies a contour specific to each view (Fig. 1). Automatic endocardial contour recognition consisted of three steps. First, a complete R-R cycle was selected from the beginning of the end-diastolic (ED) cycle to the end of the ED cycle. Second, the LV was automatically detected in the first R-wave frame of the selected cycle. Finally, view-specific deformable endocardial contour models were aligned to the individual image content. Once the endocardial boundaries were set in ED, the software automatically tracked the heart motion throughout the cardiac cycle using speckle-tracking and displayed the results of the GLS analysis (Fig. 1). The entire process, from automatic view detection to the display of analysis results, took less than 1 s. If boundary editing was required, editing in ED triggered new speckle tracking of the LV cavity boundary throughout the cardiac cycle.

Longitudinal strain (LS) was measured at the endocardial border (Fig. 1), and instantaneous strain values near the border were color-coded for visual display. In addition, changes in the strain waveform during the cardiac cycle were displayed. Segmental strain values were displayed as end-systolic strain or peak-systolic strain, and an 18-segment bull’s-eye plot showed end-systolic strain or peak-systolic strain with the time to peak (ms)^15,34^.

**Fig. 2:**
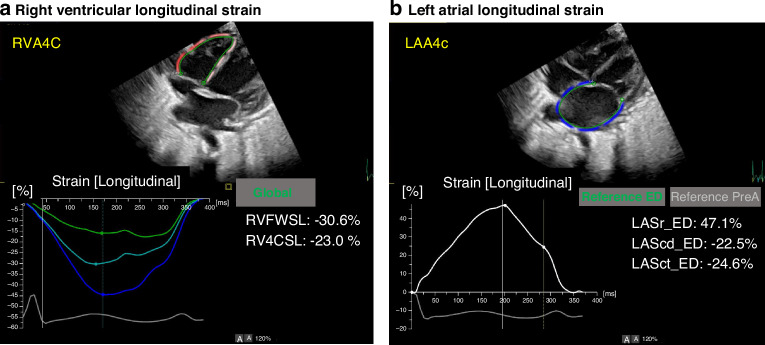
Analysis of right ventricular and left atrial longitudinal strain by speckle-tracking echocardiography. **a** Right ventricular free wall strain and four-chamber strain obtained from apical four-chamber view (RV A4C). **b** Left atrial reservoir, conduit, and contraction strain measured from LA apical view using end-diastole as reference. RV right ventricular, LA left atrial, A4C apical four-chamber view, RVFWSL right ventricular free wall longitudinal strain, RV4cSL right ventricular four-chamber longitudinal strain, LASr_ED left atrial reservoir strain with a reference time point at end-diastole, LAScd_ED left atrial conduit strain with a reference time point at end-diastole, LASct_ED left atrial contraction strain with a reference time point at end-diastole.

The same measurement of LS with automatic cross-section detection and automatic boundary detection as in the LV was also applied to the RV and LA (Fig. 2)^17^. Once the endocardial boundary was automatically placed in ED (RV) or ES (LA), it followed the heart motion using speckle tracking throughout the cardiac cycle. If the boundaries needed to be edited, they were edited in ED (LV, RV) or ES (LA). Editing the boundaries in ED (RV) or ES (LA) triggered new speckle tracking of the boundaries throughout the cardiac cycle.

Images were optimized to visualize each myocardial wall. RV systolic function was evaluated in the apical four-chamber view, which focused on the RV with all segments of the free wall and septal wall. RVFWSL and RV4cSL were evaluated using the mean of the longitudinal systolic strain peaks (Fig. 2). The software automatically generated LA myocardial surface tracings. Manual adjustments were required to optimize tracking around the LA appendage, pulmonary vein confluence, and atrial septum when necessary. To ensure accurate tracking of the atrial myocardium, the tracking performance was visually checked and the tracking geometry was further adjusted if necessary before reapplying the algorithm. Longitudinal strain of the LA was analyzed by triggering the onset of the P wave, and LASct, LAScd, and LASr were obtained from the strain curve (Fig. 2).

### Conventional transthoracic echocardiography

The following conventional echocardiographic variables were measured. We measured LV diastolic and LV systolic dimensions (mm) using the M-mode in the long-axis view. The LA and Ao diameters (mm) were measured in the long-axis view using the leading-edge method^35^. The LA area (cm^2^) and LA long-axis length (LA length, cm) were measured in the four-chamber view. The narrowest internal diameter of the PDA (mm) using 2D echocardiography was measured in the ductal long-axis view, and the PDA flow pattern was measured using the pulse wave mode (left to right, right to left, bidirectional, and none). LPAedv was measured using the pulse wave mode^13,36,37^. RV function was evaluated using the fractional area change and corrected tricuspid annular plane systolic excursion (tricuspid annular plane systolic excursion/RV long-axis diameter)^38^. LA volume was calculated using the single-plane area–length method in the four-chamber view by applying the following equation: LA volume = 0.85 × (LA area)^2^ / (LA length) (cm^3^)^13^. The LV end-systolic wall stress was calculated using mean blood pressure measurements^39,40^. Superior vena cava flow was assessed using pulse-wave Doppler as described by Kluckow and Evans^41^. The average of the maximum and minimum diameters from a still 2D image was calculated over five heart cycles. The velocity time integral was derived from Doppler velocity tracings and averaged across five consecutive cardiac cycles. Heart rate was determined from the peak-to-peak intervals of the Doppler velocity signals.

### Reproducibility analyses

Fifteen studies were randomly selected from the PDA group to investigate how measurements change over time. One observer (K. Toyoshima) measured LVGLS, RVFWSL, and LASr at 3-month intervals. The second observer (H. Aoki) who was unaware of the first measurement analyzed these data to investigate interobserver variability. We used the intraclass correlation coefficient (ICC) and Bland–Altman analysis to examine intraobserver and interobserver variabilities.

### Statistical analyses

Descriptive statistics (e.g., mean ± standard deviation, median [interquartile range]) were used to summarize the demographic or clinical data of preterm infants in the PDA and non-PDA groups. The two groups were compared using the t-test, Mann–Whitney U-test, or Fisher’s exact test. We compared hemodynamic, respiratory, and echocardiographic parameters across three time points using one-way analysis of variance with repeated measures.

Statistical analyses were conducted using EZR (version 1.54; Saitama Medical Center, Jichi Medical University, Saitama, Japan), a graphical user interface for R (The R Foundation for Statistical Computing, Vienna, Austria), along with MedCalc (version 20; MedCalc Software Ltd., Ostend, Belgium). A p value < 0.05 was regarded as statistically significant.

## Results

### Clinical data of the PDA group

**Fig. 3:**
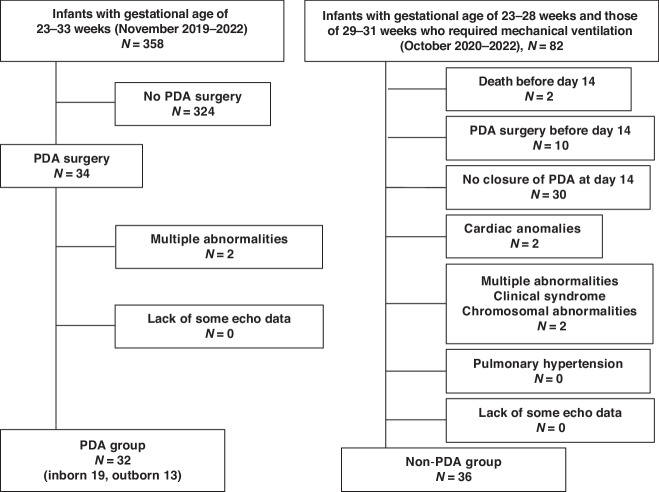
Study flow chart. In the PDA group, 32 preterm infants underwent PDA surgery at a gestational age of 23–33 weeks (November 2019 to December 2022) in our hospital. Two patients were excluded owing to the presence of multiple anomalies. Thirty-two patients were included in the final analysis, of whom 32 survived (100%). In the non-PDA group, between October 2020 and 2022, 82 inborn preterm infants were admitted to our hospital. Their gestational ages ranged from 23 to 28 weeks, and those aged between 29 and 31 weeks who required mechanical ventilation were also included. Of these, 46 patients were excluded for various reasons. As a result, 36 patients were ultimately included in the non-PDA group and 35 (97.2%) of them survived. *PDA* patent ductus arteriosus.

Thirty-two preterm infants who underwent PDA surgery were enrolled in this study between November 2019 and December 2022 (Fig. 3). The median gestational age at birth was 25 weeks (interquartile range [IQR]: 24–27), and the median birth weight was 695 g (IQR: 584–860). Pharmacological closure with cyclooxygenase inhibitors had failed in all 32 patients. PDA surgery was performed at a median age of 20 days (IQR: 15–27), with a median corrected gestational age of 28 weeks (IQR: 27–31) and a median weight of 760 g (IQR: 600–960). The median duration of surgery was 42 mi (IQR: 37–47), and the median fluid volume administered during the surgery was 27 ml/kg (IQR: 23–45). No major complications occurred in any patient during the procedure. Speckle-tracking and conventional echocardiographic measurements were successfully acquired for all preterm infants, with no missing scans. All preterm infants in this group survived to discharge.

### Reproducibility of speckle-tracking echocardiographic measurements

**Fig. 4:**
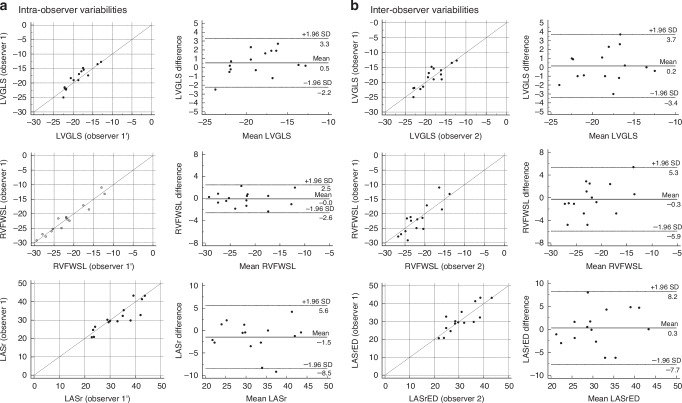
Assessment of intra- and interobserver variability in longitudinal strain measurements. **a** Intraobserver variability plots for LVGLS, RVFWSL, and LASr strain parameters. **b** Interobserver variability plots for the same parameters across two different observers. The three dashed lines show biases (means of differences) and limits of agreement. Bias is expressed as the mean of the difference (95% confidence interval). The limit of agreement is shown as the bias ± 2 SDs. LVGLS left ventricular global longitudinal strain (average), RVFWSL right ventricular free wall longitudinal strain, LASrED left atrial reservoir strain with a reference time point at end-diastole, LASctED left atrial contraction strain with a reference time point at end-diastole, SD standard deviation.

The analysis of intra- and interobserver variability in preterm infants with PDA, including the percentage bias, 95% limits of agreements, and ICCs for LVGLS, RVFWSL, and LASr, are shown in Table 1. The intrarater reproducibility (ICCs: LVGLS, 0.91; RVFWSL, 0.97; and LASr, 0.88) and interrater reproducibility (ICCs: LVGLS, 0.83; RVFWSL, 0.80; and LASr, 0.82) demonstrated very good. Figure 4 shows Bland–Altman plots, which indicated minimal bias values with acceptable limits of agreement.

**Table 1:** Intra- and interobserver variability of three-dimensional echocardiographic parameters in premature infants with patent ductus arteriosus.

Intraobserver variability			
	Bland–Altman plot		ICC (95% CI)
	Bias (95% CI)	95% LOA	
LVGLS Av	0.53 (-0.25 – 1.31)	-2.22 – 3.29	0.911 (0.757 – 0.969)
RVFWSL	-0.03(-0.74 – 0.69)	-2.56 – 2.50	0.969 (0.910– 0.990)
RV4cSL	0.05 (-1.04 – 1.14)	-3.81 – 3.91	0.884 (0.689 – 0.959)
LASr	-1.45 (-3.45 – 0.54)	-8.51 – 5.61	0.877 (0.670 – 0.96)
LAScd	0.57 (-1.81 – 2.95)	-7.84 – 8.99	0.875 (0.669– 0.956)
LASct	0.34 (-1.06 – 1.74)	-4.61– 5.29	0.904 (0.739 – 0.967)
LVGLS Av	0.15 (-0.85 – 1.16)	-3.39 – 3.70	0.826 (0.558 – 0.939)
RVFWSL	-0.29 (1.88 – 1.31)	-5.92 – 5.35	0.804 (0.510– 0.929)
RV4cSL	0.69 (-0.46– 1.83)	-3.38 – 4.75	0.852 (0.614 – 0.948)
LASr	0.29 (-1.95 – 2.54)	-7.65 – 8.24	0.821 (0.548 – 0.932)
LAScd	-2.12 (-5.69 –1.46)	-14.8 – 10.5	0.612 (0.165– 0.850)
LASct	0.86 (-1.86– 3.58)	-8.76– 10.5	0.647 (0.221 – 0.866)

ICC estimates and their 95% CIs were calculated using MedCalc® Statistical Software version 20 on the basis of a mean-rating (k = 2), absolute-agreement, two-way, mixed-effects model.

*CI* confidence interval, *ICC* intraclass correlation coefficient, *LOA* limit of agreement.

*LVGLS* left ventricular global longitudinal strain, *Av* average of A4C, A2C, and A3C measurements, *RVFWSL* right ventricular free wall longitudinal strain, *RV4cSL* right ventricular strain including the ventricular septum, *LASr* left atrial reservoir strain, *LASct* left atrial contraction strain, *LAScd* left atrial conduit strain, *BW* body weight.

### Comparison of the PDA and non-PDA groups preoperatively

Figure 3 and Tables 2 and 3 show the comparison of demographic and echocardiographic data between preterm infants in the PDA group (n = 32) and those in the non-PDA group (n = 36). All patients with PDA had a left-to-right ductal shunt. All patients had a left-to-right atrial shunt in both groups. There was no significant difference in gestational age, sex, 1- and 5-min Apgar scores, or the prevalence of small for gestational age between the groups. No significant differences were observed in corrected gestational age, body weight, or heart rate on echocardiography between the groups. However, the proportion of in-hospital birth was significantly lower in the PDA group than in the non-PDA group (p < 0.001). N-terminal prohormone brain natriuretic peptide concentrations were significantly higher in the PDA group than in the non-PDA group (p < 0.001).

**Table 2:** Comparison of demographic and echocardiographic data between the PDA group and the non-PDA group at day 14.

	PDA group	Non-PDA group	P value
Number of patients	32	36	
Gestational age (weeks)	25 (24–27)	26.5 (25–28)	0.16
Birth weight (g)	695 (5844–860)	849 (692–1045)	0.03
Male sex, n (%)	12 (37.5)	18 (50.0)	0.34
In-hospital birth, n (%)	19 (59.4)	34 (94.4)	< 0.001
Small for gestational age, n (%)	5 (15.6)	7 (19.4)	0.93
Apgar score, 1 min	4 (2–5)	4 (3–6)	0.70
Apgar score, 5 min	7 (6–8)	7 (7–8)	0.65
Cyclooxygenase inhibitor, n (%)	30 (93.8)	18 (50.0)	< 0.001
Death, n (%)	0 (0)	1 (2.8)	1
At echocardiography			
Days after birth	20 (15–27)	14	
Corrected gestational age (weeks)	28 (27–31)	29 (27–30)	0.84
Body weight (g)	760 (601–960)	858 (645–1062)	0.54

Data are presented as the mean ± standard deviation, median (interquartile range), or number (%). Student’s t-test and Fisher’s exact test were used for the comparison of continuous and categorical variables, respectively. Differences in median values between the two groups were compared using the Mann–Whitney U-test.

**Table 3:** Comparison of demographic and echocardiographic data between pre-and post-ligation in the PDA group, and between pre-ligation in the PDA group and the non-PDA group.

Parameter	Non-PDA group	PDA group				
		Preoperative	P value (vs non-PDA group)	Postoperative (4–8 h)	Postoperative (24–48 h)	P value (across 3 time points)
Hours after birth at an echocardiogram				5 (4, 6)	29 (28, 30)	
Fluid volume (ml/kg/day)	156 (145–166)	122 (110–140)*	<0.001	165 (150–180)^†^	139 (126–150)^†‡^	< 0.001
Mean airway pressure) (mmHg)	8 (8–10)	10 (8–12)	0.38	9 (8–9)	9 (8–10)	0.12
Respiratory severity score	1.9 (1.7–2.6)	2.2 (1.7–2.8)	0.99	2.1 (1.7–2.6)	2.3 (1.7–2.7)	0.25
Vasoactive–inotropic score	0 (0–0)	0 (0–0)	0.06	0 (0–0)	0 (0–0)	0.37
Catecholamine, n/N (%)	0/36 (0)	3/32 (9.3)	0.09	1/32 (3.1)	0/32 (0)	0.32
Vasodilator, n/N (%)	0/36 (0)	0/32 (0)	1	0/32 (0)	1/32 (3.1)	1
SBP (mm Hg) at an echocardiogram	68.3 ± 11.3	55.3 ± 15.1*	<0.001	51.4 ± 10.9	55.2 ± 9.0	0.068
DBP (mm Hg) at an echocardiogram	37.1 ± 8.3	28.3 ± 8.3*	<0.001	31.7 ± 7.5	31.4 ± 5.7	0.013
Heart rate (beats/minute) at an echocardiogram	156 ± 12	152 ± 14	0.36	148 ± 16^†^	144 ± 12^†^	<0.001
NT-proBNP (pg/ml)	772 (505–1460)	12004 (7963–25319)*	<0.001		3551 (2293–5685)^†^	<0.001
PDA d (mm)	0.0 ± 0.0	2.2 ± 0.8*	0.01			
LPA EDV (cm/s)	8.9 ± 1.9	25.3 ± 9.0*	<0.001	7.5 ± 4.1^†^	7.4 ± 3.0^†^	<0.001
LVDD (mm)	11.8 ± 1.6	14.4 ± 2.5*	<0 .001	11.7 ± 2.4^†^	12.7 ± 1.8^†‡^	<0.001
LVEF (M mode) (%)	64.2 ± 6.7	70.9 ± 7.7*	<0.001	58.7 ± 9.0^†^	60.1 ± 9.1^†^	<0.001
ESWS (g/cm2)	33.3 ± 9.6	34.4 ± 16.1	0.72	26.9 ± 13.4^†^	36.2 ± 13.4^‡^	<0.001
LA/Ao	1.10 ± 0.16	1.62 ± 0.18*	<0.001	1.12 ± 0.16^†^	1.23 ± 0.17^†‡^	<0.001
LAVI (ml/kg)	0.71 ± 0.23	1.74 ± 0.51*	<0.001	0.84 ± 0.27^†^	0.91 ± 0.27^†^	<0.001
LAEF (%)	56.4 ± 10.7	50.9 ± 13.3*	0.06	49.4 ± 10.7	53.2 ± 10.7	0.09
RVFAC	35.5 ± 9.8	22.3 ± 9.8*	<0.001	30.5 ± 10.8^†^	35.2 ± 11.4^†^	<0.001
cTAPSE (%)	38.0 ± 5.5	35.5 ± 6.7	0.09	30.8 ± 7.6	33.6 ± 5.8^‡^	0.02
SVCF (ml/kg/min)	191 ± 77	202 ± 79	0.61	164 ± 59^†^	172 ± 39	0.03
AutoStrain						
LVGLS 4c	-16.2 ± 2.7	-19.6 ± 3.4*	<0.001	-13.5 ± 3.6^†^	-15.8 ± 3.5^†‡^	<0.001
LVGLS 2c	-15.9 ± 3.0	-21.0 ± 3.7*	<0.001	-13.4 ± 3.7^†^	-15.3 ± 3.6^†‡^	<0.001
LVGLS 3c	-15.0 ± 2.3	-18.5 ± 3.8*	0.016	-12.1 ± 3.6^†^	-14.3 ± 2.8^†‡^	<0.001
LVGLS Av	-15.7 ± 2.3	-19.7 ± 3.3*	<0.001	-12.9 ± 3.5^†^	-15.1 ± 3.0^†‡^	<0.001
RVFWSL	-27.6 ± 5.3	-21.3 ± 4.7*	<0.001	-20.7 ± 6.9	-24.7 ± 6.0^†‡^	<0.001
RV4cSL	-22.7 ± 4.0	-16.6 ± 7.5	0.081	-15.4 ± 9.7	-20.6 ± 3.9^†‡^	<0.001
LASr	34.8 ± 8.6	31.2 ± 7.8	0.28	24.5 ± 10.3^†^	31.4 ± 9.7^‡^	0.003
LAScd	-22.1 ± 7.8	-20.1 ± 7.1	0.36	-16.4 ± 8.3	-17.8 ± 11.3	0.132
LASct	-12.7 ± 7.2	-11.0 ± 7.9*	0.04	-8.0 ± 7.4	-10.5 ± 8.4	0.040

Data are presented as the mean ± standard deviation, median (interquartile range), or number (%) unless shown otherwise.

Differences between the two groups were analyzed using the unpaired t-test for continuous variables, Mann–Whitney U-test for median values, and Fisher’s exact test for categorical data. Hemodynamic, respiratory, and echocardiographic parameters were compared across the three time points using one-way analysis of variance with repeated measures.

*P < 0.05, versus the non-PDA group and preoperatively in the PDA group; ^**†**^P < 0.05, versus preoperatively; ^**‡**^P < 0.05, versus 4–8 h after surgery.

NT-proBNP values represent measurements taken at three time points: (1) on day 14 in the non-PDA group, (2) within 12 h preoperatively in the PDA group, and (3) within 24–48 h postoperatively in the PDA group.

*NT-proBNP* N-terminal pro-brain natriuretic peptide, *PDA* patent ductus arteriosus, *LPA* left pulmonary artery, *EDV* end-diastolic velocity, *LVDD* left ventricular diastolic dimension, *LVEF* left ventricular ejection fraction, *ESWS* end-systolic wall stress, *LA/Ao* left atrial diameter to aortic diameter ratio, *LAVI* left atrial volume index, *LAEF* left atrial emptying fraction, *RVFAC* right ventricular fractional area change, c*TAPSE* corrected tricuspid annular plane systolic excursion, *SVCF* Doppler volumetric measurements of superior vena cava flow, *LVGLS* left ventricular global longitudinal strain, *A4c* apical four-chamber view, *A2c* apical two-chamber view, *A3c* apical three-chamber view, *Av* average of A4C, A2C, and A3C measurements, *RVFWSL* right ventricular free wall longitudinal strain, *RV4cSL* right ventricular strain including the ventricular septum, *LASr* left atrial reservoir strain, *LASct* left atrial contraction strain, *LAScd* left atrial conduit strain, *BW* body weight.

Pre-ligation blood pressure was measured using an arterial line in 21 of 32 (66%) preterm infants. In the remaining 11 of 32 (34%) preterm infants in the PDA group and in all of those in the non-PDA group, blood pressure was assessed using the oscillometric technique. Systolic and diastolic blood pressure were significantly lower in the PDA group than in the non-PDA group (both p < 0.001).

The PDA group had a significantly larger LVEF and LVGLS than the non-PDA group (both p < 0.001) (Table 3).

In contrast, the PDA group had less RV wall motion indicated by a significantly smaller RVFWSL, and lower corrected tricuspid annular plane systolic excursion and RV fractional area exchange than the non-PDA group (Table 3). However, the difference in RV4cSL between the groups was not significant (p = 0.081).

The indices of LA enlargement (LA/Ao, LA volume) in the PDA group were higher than those in the non-PDA group (both p < 0.001). The LAEF and LASr were not significantly different between the groups, but LASct was lower in the PDA group than in the non-PDA group (p = 0.04) (Table 3).

### Comparison of pre-ligation, 4–8 h post-ligation, and 24–48 h post-ligation in the PDA group

Heart rate at 4–8 and 24–48 h after surgery was significantly lower than that preoperatively (both p < 0.05). LVDD was also significantly decreased at 4–8 and 24–48 h postoperatively compared with that preoperatively (both P < 0.001). Additionally, the LPAedv, LVEF, LA/Ao ratio, and LA volume index were significantly reduced postoperatively compared with preoperatively (all p < 0.001) (Table 3).

**Fig. 5:**
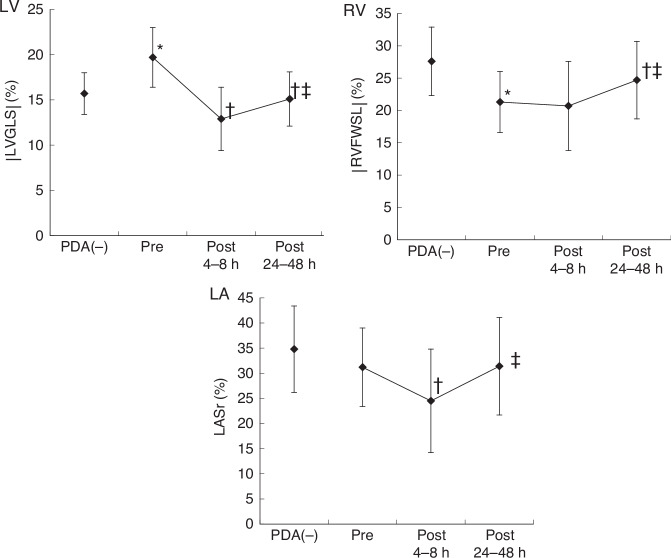
Comparison between non-PDA, pre-ligation, post-ligation at 4–8 h, and post-ligation at 24–48 h. Changes in LVGLS, RVFWSL, and LASr across four time points: non-PDA group, pre-ligation, 4–8 h post-ligation, and 24–48 h post-ligation. Error bars represent mean ± SD. Statistical annotations: *p < 0.05 vs. non-PDA; †p < 0.05 vs. pre-ligation; ‡p < 0.05 vs. 4–8 h post-ligation. *|LVGLS|* absolute value of left ventricular global longitudinal strain (average of A4C, A2C, and A3C), *|RVFWSL|* absolute value of right ventricular free wall longitudinal strain, *LASr* left atrial reservoir strain, *PDA(-)* non-PDA, *PDA* patent ductus arteriosus.

LVGLS was significantly decreased at 4–8 h (p < 0.001) and slightly increased at 24–48 h (p < 0.01) postoperatively compared with preoperatively (Fig. 5 and Table 3). RVFWSL and RV4cSL were increased at 24–48 h postoperatively compared with preoperatively (both p < 0.001) (Fig. 5 and Table 3), without a reduction at 4–8 h postoperatively. LASr was significantly decreased at 4–8 h and slightly increased at 24–48 h postoperatively compared with preoperatively (both p < 0.01) (Fig. 5 and Table 3).

## Discussion

The present study provides a detailed strain analysis, including phase-specific LA and RV in addition to LV, which were calculated using the highly adaptable AutoStrain software powered by artificial intelligence. To the best of our knowledge, this is the first study to show RVFWSL in this population. This strain approach allows a more comprehensive understanding of the loading and functional status, which dramatically changes after PDA surgical closure, and may provide insight into optimizing individual treatment in a tailored fashion in these preterm infants^40^.

### Comparison of the PDA and non-PDA groups preoperatively

In our study, LV, RV, and LA volumes were successfully quantified by speckle-tracking echocardiography in preterm infants with and without PDA. Significant differences in LVGLS and RVFWSL values were observed between the PDA and non-PDA groups. The absolute values of LVGLS, RVFWSL, and LASct in the PDA group were 125%, 77%, and 87% of those in the non-PDA group, respectively. In the PDA group, LVGLS was increased, reflecting excessive preload and reduced afterload. In contrast, preoperative RVFWSL was reduced. This observation may be attributed to several factors, such as increased RV preload due to a left-to-right shunt via a stretched foramen ovale caused by an enlarged LA by PDA shunt, increased RV afterload due to elevated LA and pulmonary arterial pressure, and decreased coronary flow induced by an increased aorta-to-pulmonary artery shunt via PDA.

Regarding the LA, there was no significant difference in LASr or LAScd between the PDA and non-PDA groups preoperatively. Only LASct was lower in the PDA group than in the non-PDA group. Although the LA enlarges as PDA becomes more severe, LA strain does not necessarily decrease, possibly due to compensatory mechanisms.

### Comparison of pre-ligation, 4–8 h post-ligation, and 24–48 h post-ligation

In this study, LVGLS and LASr were significantly decreased 4–8 h postoperatively. This decrease is likely due to the increased LV afterload after PDA ligation and the sudden reduction in LV and LA volumes^14^. By 24–48 h after surgery, LVGLS, LASr, and LAScd had returned to the levels similar to those of preterm infants without PDA. The recovery of LVGLS and LASr between 4–8 and 24–48 h after surgery may reflect compensatory mechanisms for increased afterload after surgery, which include recovery of the volume status and that from the effects of anesthesia.

However, LASct remained low in the PDA group, suggesting that recovery of LA contractile function may take longer even after PDA-induced LA enlargement. In contrast to the LVGLS, RVFWSL did not decrease 4–8 h after surgery and actually increased 24–48 h after surgery to the level of that in the non-PDA group. This finding suggests that RV dysfunction associated with PDA may rapidly improve owing to a decrease in LA pressure and loss of the PDA shunt to the pulmonary vasculature. However, severe RV dysfunction in premature infants with PDA may be another sign of severity of PDA that has not been focused on to date.

These postoperative changes in LVGLS, LASr, and RVFWSL strain may serve as useful indices for postoperative management of the circulation. They may help determine and predict the pathogenesis of post-ligature syndrome.

### Demonstrating the reliability of speckle-tracking echocardiography in serial assessments before and after PDA ligation

We showed the reliability of speckle-tracking echocardiography in serial assessments before and after PDA ligation. Assessment of LV, LA, and RV function using the speckle-tracking technique has recently become a standard clinical tool for evaluating cardiac function in adults. LVGLS decreases before the LVEF, which reflects potential LV systolic dysfunction in heart failure with a preserved LVEF^15^. LVGLS is an important predictive marker of early cardiac dysfunction, and LVGLS is also an independent prognostic factor in heart failure with a reduced LVEF^42^. LASr can be used to evaluate reservoir function, which stores blood during systole by relaxation and extension of the LA, and the booster function, which ejects stored blood to the LV during late diastole^20,21^.

The assessment of LV, LA, and RV longitudinal strain using speckle-tracking echocardiography has only been sporadically reported in preterm infants^16,22–30^. RVFWSL is considered more important in assessing RV function because it excludes the intraventricular septum, which is strongly affected by LV function^17,19^. RVFWSL is used for diagnosing and predicting the prognosis of right heart failure and pulmonary hypertension^18,19^.

The study confirmed the robustness of speckle-tracking echocardiography in detecting changes in cardiac function, and showed high intra- and interobserver reliability. The AutoStrain application uses the automation technologies Auto View Recognition and Auto Contour Placement. The implementation of these automation tools provides a simple, fast workflow for robust and reproducible longitudinal strain measurements. However, editing and overriding this automation could make AutoStrain a convenient, non-invasive and useful echocardiographic indicator in clinical practice. We have reported that echocardiography-based tailored management of PDA in preterm infants may save lives with fewer complications^40,43^. Speckle-tracking echocardiography using artificial intelligence (machine learning) techniques may be useful in understanding the hemodynamics of PDA in preterm infants.

Finally, this study provides novel insights into cardiac adaptation after PDA ligation using machine learning-based speckle-tracking echocardiography. While our previous study evaluated biventricular volume and function using 3D echocardiography^14^, this study extends this analysis to myocardial deformation, offering a more comprehensive assessment of ventricular and atrial mechanics. Notably, to the best of our knowledge, this is the first study to comprehensively assess LA longitudinal strain in each phase (LASct, LAScd, and LASr), as well as RVFWSL and RV4cSL. These parameters provide crucial insights into how preterm infants with severe PDA and those without PDA differ in myocardial strain, and they were not measured in prior volume-based assessments. Although our previous study reported LVGLS using 3D echocardiography, the lower frame rate and different computational principles limit direct comparisons with 2D speckle-tracking echocardiography. In contrast, AutoStrain enables a high frame rate and standardized strain analysis, making it a clinically relevant feasible tool for assessing myocardial deformation in preterm infants.

### Study limitations

This study has several limitations. First, because of the small sample size and the absence of patients who developed post-ligation instability syndrome, we were unable to assess the effect of speckle-tracking methods on clinical outcomes compared with traditional echocardiographic assessment. Nevertheless, we successfully obtained continuous hemodynamic data after PDA ligation in preterm infants, and captured the acute (4–8 h) and subacute (24–48 h) phases using semi-automated measurements with machine learning algorithms.

Second, this study was conducted at a single center, and there is substantial variability in the evaluation and management of preterm infants with PDA across different centers and countries. Postoperative outcomes may widely vary depending on preoperative conditions, the management approach, and anesthesia during surgery. Therefore, the generalizability of these findings should be considered with caution.

The echocardiographer had access to the patients’ clinical information and background data during the data extraction process, which could have introduced potential bias. Nevertheless, the extraction procedures were semi-automated and the inter- and intraobserver variability was within acceptable limits. Notably, only one vendor’s algorithm was used.

## Conclusions

Speckle-tracking echocardiography shows the different functional abnormalities and post-surgical adaptation processes in LVGLS, LASr, and RVFWSL in preterm infants requiring a PDA ligation. Notably, RVFWSL normalized within 24–48 h after surgery, while LVGLS and LASr showed partial recovery. Future prospective studies are required to determine whether assessment of LVGLS, RVFWSL, and LASr using speckle-tracking echocardiography could help to optimize tailored management and improve outcomes in this patient population.

## Data Availability

The data underlying the findings of this study can be accessed upon reasonable request by contacting the corresponding author (K. Toyoshima).
